# Airway Exosomes Released During Influenza Virus Infection Serve as a Key Component of the Antiviral Innate Immune Response

**DOI:** 10.3389/fimmu.2020.00887

**Published:** 2020-05-12

**Authors:** James G. Bedford, Giuseppe Infusini, Laura F. Dagley, Fernando Villalon-Letelier, Ming Z. M. Zheng, Vicki Bennett-Wood, Patrick C. Reading, Linda M. Wakim

**Affiliations:** ^1^Department of Microbiology and Immunology, The University of Melbourne, The Peter Doherty Institute for Infection and Immunity, Melbourne, VIC, Australia; ^2^Department of Medical Biology, The Walter and Eliza Hall Institute of Medical Research, The University of Melbourne, Melbourne, VIC, Australia; ^3^WHO Collaborating Centre for Reference and Research on Influenza, Victorian Infectious Diseases Reference Laboratory, The Peter Doherty Institute for Infection and Immunity, Melbourne, VIC, Australia

**Keywords:** influenza, exosomes, antiviral activity, mucosal immunity, airway inflammation

## Abstract

Exosomes are extracellular vesicles secreted by cells that have an important biological function in intercellular communication by transferring biologically active proteins, lipids, and RNAs to neighboring or distant cells. While a role for exosomes in antimicrobial defense has recently emerged, currently very little is known regarding the nature and functional relevance of exosomes generated *in vivo*, particularly during an active viral infection. Here, we characterized exosomes released into the airways during influenza virus infection. We show that these vesicles dynamically change in protein composition over the course of infection, increasing expression of host proteins with known anti-influenza activity, and viral proteins with the potential to trigger host immune responses. We show that exosomes released into the airways during influenza virus infection trigger pulmonary inflammation and carry viral antigen that can be utilized by antigen presenting cells to drive the induction of a cellular immune response. Moreover, we show that attachment factors for influenza virus, namely α2,3 and α2,6-linked sialic acids, are present on the surface of airway exosomes and these vesicles have the ability to neutralize influenza virus, thereby preventing the virus from binding and entering target cells. These data reveal a novel role for airway exosomes in the antiviral innate immune defense against influenza virus infection.

## Introduction

Exosomes are small vesicles (30–100 nm in diameter) of endocytic origin that are released from cells into the extracellular environment during normal and pathological conditions (1). They are formed by the inward budding of late endosomal membranes that give rise to intracellular multivesicular bodies (MVBs) which fuse with the plasma membrane releasing the intraluminal exosomes into the extracellular space. They are secreted by virtually all cell types and are present in bodily fluids such as blood, urine, saliva, breast milk, bronchial, and nasal lavage (2–6). Although the protein composition of exosomes reflects that of the parent cell, exosomes are generally rich in tetraspanins (CD9, CD63, CD81), heat shock proteins and Rab proteins. Exosomes are an important tool for intercellular communication through the transfer of biologically active proteins, lipids, and RNAs (7, 8). Only recently has a role for exosomes in viral pathogenesis and antimicrobial defense emerged.

*In vitro* studies have demonstrated that exosomes play a dual role, promoting pathogen transmission, and exacerbation of infection in some instances but contributing to host defense and control of infection in others. For example, exosomes released from cells infected with an array of RNA viruses including human immunodeficiency virus, hepatitis C virus, human T-cell lymphotropic virus and dengue virus, carry viral proteins and RNA and these vesicles can facilitate virion-independent transfer of replication-competent virus between cells (1, 9). In this context, exosomes facilitate the spread of the virus. However, exosomes can also limit the spread of virus through a variety of mechanisms. Exosomes isolated from certain cell lines can express an array of interferon (IFN)-induced antiviral proteins which interfere with viral replication and enhance the ability of uninfected cells to resist infection (10). Exosomes serve as a method for the intercellular transfer of these proteins, conferring broad-spectrum viral resistance. Exosomes generated in the presence of type I IFN and expressing an array of antiviral molecules could render cells resistant to *in vitro* infection by hepatitis B virus (11) or Dengue virus (12). Furthermore, exosomes recovered from human respiratory epithelial cell lines have also been reported to bind and neutralize human influenza virus (13). To date, nearly all data supporting the antiviral activity of exosomes has been derived from *in vitro* studies where exosomes were derived from cultured cell lines. Currently, very little is known regarding the characteristics of exosomes generated *in vivo*, how they might change during infection and whether they contribute to the antiviral immune response.

Here we profiled exosomes released into the airways of mice over the course of an influenza virus infection. Proteomic analysis of these vesicles revealed temporal changes in protein composition, with exosomes gaining host proteins with known anti-influenza activity, as well as viral proteins with the potential to trigger host immune responses. This altered protein composition afforded these vesicles new capabilities allowing them to trigger pulmonary inflammation and serve as a source of viral antigen that could be utilized for the induction of a cellular immune response. We also show that attachment factors for influenza virus, namely α2,3 and α2,6-linked sialic acids, are present on the surface of airway exosomes and these vesicles have the ability to neutralize influenza virus, thereby preventing the virus from binding and entering target cells. These data highlight a variety of biological functions by which exosomes released into the airways during an influenza virus infection assist in the antiviral innate immune response.

## Results

### Airway Exosomes Dynamically Change in Protein Composition Over the Course of an Influenza Virus Infection

**Figure 1:**
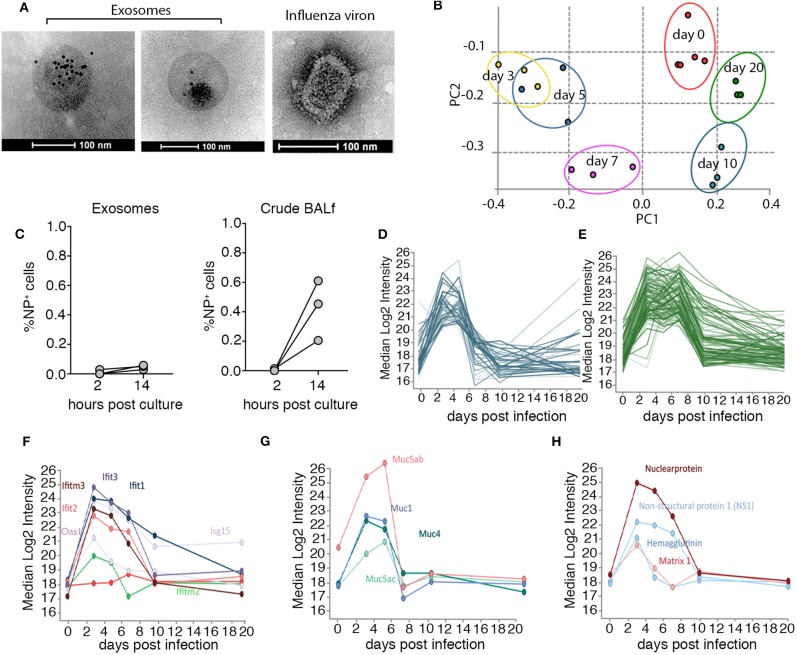
Airway exosomes dynamically change in protein composition over the course of an influenza virus infection. **(A)** Electron microscope images of CD9-gold stained exosomes recovered from the BALf of mice on day 3 pi and an influenza viron used as a control to demonstrate differences in morphology. **(B)** 0.75 μg of purified exosomes or matched amounts of crude BALf recovered from mice 2 days post intranasal influenza virus infection were inoculated onto MDCKs and the proportion of influenza virus nucleoprotein (NP) positive cells 2 and 14 h later was measured by flow cytometry. Data pooled from 3 independent experiments. **(C)** Principal component analysis demonstrates the clustering of biological replicates and the restoration of the proteome to an uninfected like state by day 20. **(D,E)** Median Log2 Intensity of the proteins that increase in abundance over the acute phase of infection **(F–H)** clustered proteins based on elevated expression over acute phase of infection **(F)** antiviral proteins **(G)** mucins **(H)** influenza virus proteins.

To gain insight into the nature of exosomes generated *in vivo*, we optimized protocols to isolate exosomes from murine bronchial alveolar lavage fluid (BALf) and profiled the protein composition of these vesicles at different time-points over the course of influenza virus infection using mass spectrometry. Mice infected with influenza virus via the intranasal route were euthanized at various times post-infection and BALf was collected, filtered (0.2 μm) and exosomes were purified by ultra-centrifugation (14) followed by further enrichment by immune-absorption onto magnetic beads coated with antibodies against the tetraspanin CD9, a common exosomal marker (15) (Supplementary Figure 1). The preparation resulted in the isolation of highly pure exosome preparations. To confirm exosome preparations were free from influenza virion contamination we stained the vesicles with anti-CD9 gold-labeled particles and visualized the samples using electron microscopy. We failed to identify any influenza virions, which can be easily discriminated from exosomes based on morphology, in our purified exosome preparations (Figure 1A). Moreover, when we cultured purified airway exosomes recovered from mice on day 2 post influenza virus infection on MDCKs, a canine kidney cell line that is highly permissive to influenza virus infection, we did not observe any overt infection, as measured by an increase in influenza virus nucleoprotein (NP) staining, implying that there were no infectious virions in our exosome preparation or the level present was below our limit of detection (Figure 1B). We next performed semi-quantitative proteomics analysis on exosome preparations which resulted in the identification of 2,688 proteins. To confirm the exosome preparations were pure we firstly checked our dataset for a panel of classic exosome markers and found that these were present and highly abundant in our samples, whereas protein markers for other cellular compartments (i.e., ER or Golgi) were rare (Supplementary Figure 2 and Supplementary Table 1). Principle components analysis of the exosome samples revealed striking differences in the protein composition of these vesicles over the course of the influenza virus infection (Figure 1C). Moreover, the effect was enduring, as even at day 20 post influenza infection the protein composition of airway exosomes failed to return to the baseline composition of exosomes recovered from naïve animals. To identify proteins elevated in the exosomes in response to influenza virus infection we refined our analysis to proteins that increased in abundance over the acute phase (day 3–7) of infection (Figures 1C,D and Supplementary Tables 2, 3). Interestingly, many antiviral proteins with known anti-influenza activity, including members of the IFIT and IFITM families, were present at high levels in exosomes recovered from mice during the acute phase of infection (Figure 1F). In addition, expression levels of proteins of the mucin family were also markedly enhanced on exosomes during the acute phase of infection. In previous studies, exosomes recovered from respiratory epithelial cell lines were shown to express sialylated mucins and these vesicles could bind and neutralize influenza virus (13). In addition, four influenza virus proteins (HA, NS1, NP, and M1) were identified in airway exosomes (Figure 1G). Together, these data confirm the presence of host proteins with known anti-influenza activity, and viral proteins with the potential to trigger host immune responses, in exosomes derived from the airways of influenza virus-infected mice.

### Airway Exosomes From Influenza Virus Infected Mice Evoke Pulmonary Inflammation

**Figure 2:**
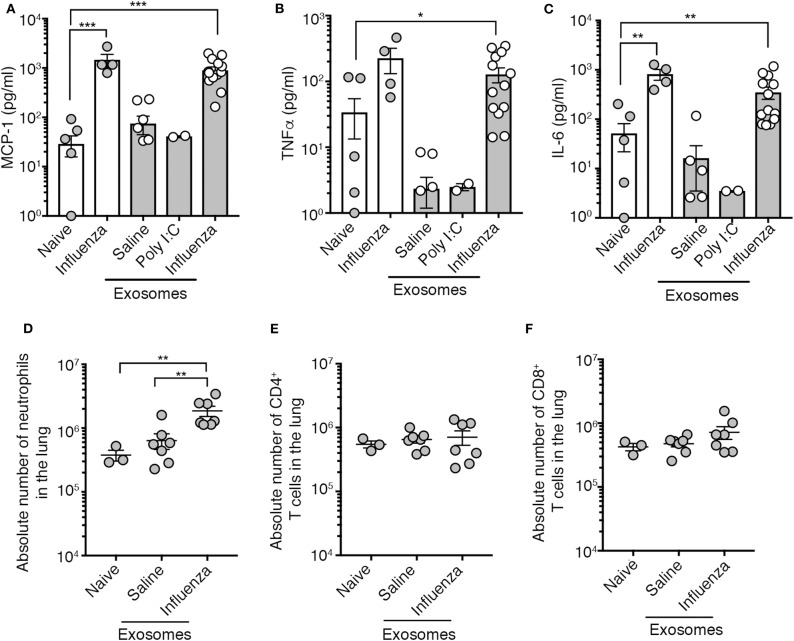
Airway exosomes from influenza virus-infected mice evoke airway inflammation. **(A–C)** Levels of inflammatory cytokines in mouse BALf. Mice that received 0.75 μg of airway exosomes recovered from mice 2 days after intranasal delivery of either saline, poly I:C or influenza virus were killed 4 days later for analysis of inflammatory cytokines in BALf. In these studies, BALf of naïve mice and from mice infected 4 days earlier with influenza virus were included as controls. Data pooled from 2 to 3 experiments. Symbols represent individual mice (*n* = 2–14 mice per group, one-way ANOVA Dunnett's multiple comparison). **(D–F)** The absolute number of neutrophils (Ly6g+CD11b+), CD4+ T cells and CD8+ T cells in the lung of mice 4 days following intranasal delivery of 0.75 μg of exosomes recovered from the BALf of mice 2 days after intranasal delivery of either saline or influenza virus. Data pooled from 3 independent experiments. Symbols represent individual mice (*n* = 3–7 mice per group, one-way ANOVA, Tukey's multiple comparison). *p < 0.05, **p < 0.01, ***p < 0.001.

We tested the capacity of exosomes recovered from the BALf of influenza virus-infected mice to trigger pulmonary inflammation. To this end, we intranasally transferred into the airways of naïve mice an equal amount of exosomes recovered from the BALf of either naïve mice, mice infected 2 days prior with influenza virus, or mice given an inflammatory agent (poly I:C). Four days following exosome delivery we assessed the level of inflammation by measuring cytokine levels in the BALf and immune cell infiltration into the lung tissue. As a positive control for pulmonary inflammation, we also included a cohort of mice directly infected with influenza virus. The delivery of exosomes recovered from the airways of influenza virus-infected mice resulted in the production of IL-6, MCP-1 and TNF, which was a very similar inflammatory profile to that observed following direct infection with influenza virus (Figures 2A–C). Widening our analysis to include assessment of a more extensive panel of inflammatory cytokines revealed that exosomes recovered from the airways of influenza virus-infected mice also resulted in the production of both type I and type II interferon (Supplementary Figure 3). In contrast, the intranasal delivery of exosomes recovered from naïve or poly I:C treated mice did not evoke the release of any cytokines (Figures 2A–C), implying that the PAMPs or potential DAMPs loaded into the exosomes generated during virus infection were essential to trigger inflammation. Exosomes derived from influenza virus infected mice did contain viral RNA which may serve as the PAMP triggering the observed inflammatory response (Supplementary Figure 4). Consistent with the capacity to trigger the release of inflammatory cytokines, the intranasal delivery of exosomes recovered from the BALf of influenza virus-infected mice, but not naïve mice also resulted in the recruitment of neutrophils while the number of CD8^+^ and CD4^+^ T cells remained stable irrespective of the treatment (Figures 2D–F). Thus, exosomes released into the airways during an influenza virus infection are inflammatory, causing the release of cytokines/chemokines and resulting in the recruitment of innate immune cells.

### Airway Exosomes Contain Influenza Virus Antigen Capable of Serving as a Source of Antigen in the Initiation of a Cellular Immune Response

**Figure 3:**
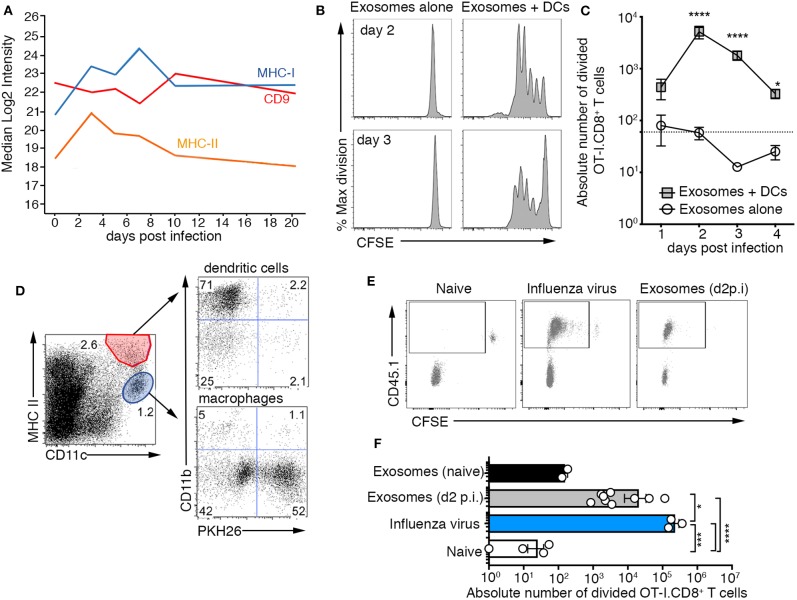
Airway exosomes contain influenza virus antigen and are capable of initiating a cellular immune response. **(A)** Proteomic analysis measuring levels of CD9, MHC II, CD74, and MHC I expressed by exosomes isolated from BALf over the course of an influenza virus infection. **(B,C)** Exosomes purified from the BALf of mice infected 1–4 days prior with 10^4^ PFU of X31-OVA (1) were cultured with 5 × 10^4^ CFSE-labeled OT-I CD8^+^ T cells with or without 1 × 10^4^ DCs. T cell proliferation was measured 60 h later. **(B)** Representative OT-I.CD8+ T-cell proliferation profiles with exosomes recovered from the BALf on days 2 and 3 p.i., cultured with or without DCs. **(C)** The absolute number of divided OT-I.CD8+ T cells. Data pooled from 3 to 9 independent experiments. Symbols represent the mean ± sem (two-way ANOVA, Sidak's multiple comparison). Dotted line represents the background level of T-cell division. **(D)** PKH26-labeled exosomes were intranasally administered into mice and 1 h later the lungs were recovered and the proportion of DCs and macrophages that had engulfed PKH67+ exosomes was profiled. **(E)** Mice injected with 2 × 10^6^ CFSE-labeled OT-I.CD8^+^ T cells were intranasally administered either saline (naïve) or x31-OVA (1) or 0.75 mg of exosomes recovered from the BAL of naïve mice or mice infected with x31-OVA (1) 2 days earlier and the absolute number of divided OT-I cells in the mLN was measured 4 days later. **(E)** Representative flow cytometry profiles gated on CD8+ T cells. **(F)** Graph depicts the absolute number of divided cells. Data pooled from 4 independent experiments (one-way ANOVA, Dunnett's multiple comparison). *p < 0.05, ***p < 0.001, ****p < 0.0001.

Previous studies have reported a role for exosomes in antigen presentation demonstrating that they can deliver immunologically relevant proteins/peptides to antigen presenting cells which in turn can utilize these antigens to initiate a T cell response. As exosomes released into the airways during influenza virus infection carried a number of viral proteins (Figure 1H), as well as major histocompatibility class I and II complexes (Figure 3A), we next assessed whether these vesicles could initiate CD8^+^ and CD4^+^ T cell activation. To analyze antigen-specific, MHC-restricted T cell stimulation by exosomes, we infected mice with a recombinant influenza virus engineered to express the CD8 (SIINFEKL, x31-OVA_1_) (16) epitope from the model antigen Ovalbumin (OVA). Then, on days 1–4 post-infection, exosomes recovered from the BALf were cultured with carboxyfluorescein diacetate succinimidyl ester (CFSE) labeled OVA-specific OT-I.CD8^+^ T cells with or without dendritic cells. Irrespective of the time point at which they were recovered, exosomes failed to drive OT-I.CD8^+^ T cell proliferation if dendritic cells were not added to the cultures (Figures 3B,C). In contrast, CD8^+^ T cell proliferation was observed when exosomes and dendritic cells were present in the cultures, implying that airway exosomes can act as a source of antigen but cannot present CD8^+^ T cell epitopes directly. Dendritic cells fed exosomes recovered from the BALf at day 2–3 p.i. were capable of driving the most robust T cell division, which is consistent with our proteomic analysis demonstrating the viral antigen load in the exosomes peaked < day 3 p.i. and declined thereafter. We also checked the capacity of exosomes to drive MHC-II presentation, by infecting mice with an influenza virus that expressed the CD4^+^ OVA epitope (ISQAVHAAHAEINEAGR; x31-OVA_2_) (17), harvesting exosomes from the BALf 2 days later and culturing these vesicles *in vitro* with CFSE labeled OVA specific CD4^+^ T cells, once again, with or without dendritic cells. Consistent with our findings evaluating MHC-I presentation, we find that CD4^+^ T cell proliferation was observed only in the presence of dendritic cells (Supplementary Figure 5A). Together, these findings imply that exosomes cannot stimulate T cell activation directly but instead can act as a source of antigen.

We next tested whether exosomes released into the airways during influenza virus infection could deliver antigen to antigen presenting cells, *in vivo*. First, we checked whether antigen presenting cells could capture airway exosomes. To this end, we transferred PHK26-dye labeled exosomes intranasally into mice and 1 h later showed that while both macrophages and dendritic cells in the lung had engulfed these fluorescent vesicles, the vast majority were associated with macrophages (Figure 3D). To test the capacity of airway exosomes to serve as a source of antigen *in vivo*, we transferred allelically tagged (CD45.1^+^) CFSE labeled OT-I.CD8^+^ T cells into C57BL/6 (CD45.2^+^) mice, followed by intranasal delivery of exosomes recovered from the BALf of naïve mice or mice infected 2 days prior with x31-OVA_1_. As controls, additional cohorts of OT-I.CD8^+^ T cell recipient mice were left naïve or were directly infected with x31-OVA_1_. OT-I.CD8^+^ T cell proliferation in the lung-draining lymph node (mediastinal lymph, mLN), measured as a loss of CFSE dye, was assessed 4 days later. As expected, OT-I.CD8^+^ T cells remained undivided (CFSE^hi^) in naïve animals and underwent multiple rounds of divisions in animals directly infected with x31-OVA_1_. While OT-I.CD8^+^ T cells remained undivided in animals that received exosomes from naïve mice, significant T-cell division was observed in animals that received exosomes recovered from the BALf of influenza virus-infected animals (Figures 3E,F). Similar results were observed when we used exosomes from the BALf of x31-OVA_2_ infected mice and tracked OT-II.CD4^+^ T division *in vivo* (Supplementary Figure 5B). Collectively this data shows that exosomes released into the airways during influenza virus infection carry viral antigen that can be utilized by antigen presenting cells to drive both CD4^+^ and CD8^+^ T cell responses.

### Airway Exosomes Generated Following Infection With a Replication Incompetent Virus Evoke Pulmonary Inflammation and Serve as a Source of Antigen for the Development of a Cellular Immune Response

**Figure 4:**
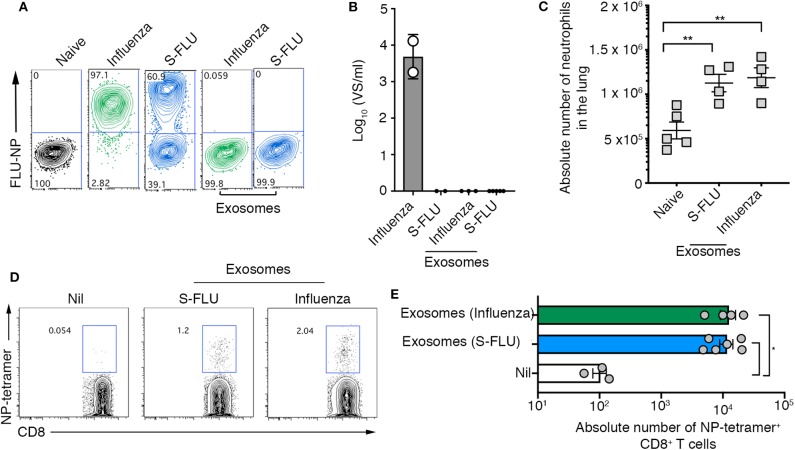
Airway exosomes generated following infection with a replication incompetent virus evoke inflammation and influenza virus-specific cellular immune responses. **(A,B)** MDCK cells were infected in the presence of trypsin at a multiplicity of infection (moi) of 10 with influenza virus or a S-FLU, or were cultured with 0.75 μg of exosomes purified from the BALf of mice infected with 2 days earlier with S-FLU. The proportion of influenza virus NP+ cells was determined 14 hrs later by flow cytometry. Data representative of 2 independent experiments. **(B)** Titres of infectious influenza virus released from virus-infected cells were quantitated using a virospot assay. Bars represent the mean + sd. **(C)** Absolute numbers of neutrophils (Ly6g^+^CD11b^+^) in the lung of mice 4 days following intranasal delivery of 0.75 μg of exosomes recovered from BALf of mice 2 days after intranasal delivery of either influenza virus (x31) or S-FLU. Data pooled from 2 independent experiments. Symbols represent individual mice (*n* = 2–5 mice per group, one-way ANOVA, Tukey's multiple comparison). **(D,E)** Mice received 0.75 μg of exosomes recovered from the BALf of mice infected with either influenza virus (x31) or S-FLU 2 days earlier and the absolute number of influenza virus nucleoprotein-specific cells (NP-tetramer+) in the mLN was measured 7 days later. **(D)** Representative flow cytometry profiles gated on CD8^+^ T cells. **(E)** Graph depicts the absolute number of divided CD8+ NP-tetramer+ cells. Data pooled from 2 independent experiments (one-way ANOVA, Dunnett's multiple comparison). *p < 0.05, **p < 0.01.

Here we show that exosomes released into the airways of influenza virus infected mice dynamically change in protein composition, are capable of evoking airway inflammation, and contain viral proteins that can be utilized by dendritic cells to drive both CD4^+^ and CD8^+^ T cell responses. While we optimized protocols to ensure that our exosome preparations were free of influenza virions, the separation of enveloped viruses from extracellular vesicles is challenging due to the similarity of these bio-nanoparticles. Hence, we performed a series of experiments utilizing replication incompetent influenza virus strains to confirm that the findings we report were not merely due to the presence of contaminating influenza virus particles. We utilized a non-replicating influenza virus which was inactivated by suppression of the haemagglutinin signal sequence (S-FLU) and therefore can only infect for a single round (18). To confirm the replication defective nature of the S-FLU virus we infected MDCKs with either wild-type influenza virus (x31), or S-FLU, in the presence of exogenous trypsin to facilitate multiple cycles of virus replication and measured virus infection 18 h later, by assessing the proportion of cells staining positive for influenza virus NP. The majority of cells directly infected with the S-FLU variant stained positive for viral NP, confirming that this virus can infect cells and, importantly, synthesize influenza virus proteins within infected cells. However, we could not detect any infectious influenza virus released into the supernatant of these cultures. Together, these findings validate the capacity of the S-FLU virus to undergo only a single round of replication within infected cells without production of infectious virus progeny (Figures 4A,B). In contrast, cells infected with the wild-type influenza virus stained positive for influenza NP and culture supernatants contained high levels of infectious virus (Figures 4A,B).

Next, purified airway exosomes recovered from BALf of mice 2 days after infection with wild-type or S-FLU were cultured with MDCK cells in the presence of exogenous trypsin to facilitate multiple cycles of virus replication and the proportion of virus-infected cells, as well as the titres of virus released into the culture supernatant, were determined. While exosomes recovered from the BALf of wild-type virus-infected mice resulted in negligible levels of NP staining in MDCK cells, no NP staining was observed following the culture of exosomes recovered from the BALf of S-FLU infected mice. Moreover, supernatants recovered from the cultures of MDCK cells cultured with exosomes purified from BALf of mice infected with wild-type virus or S-FLU did not contain detectable levels of infectious virus implying that there were no infectious virions in either of the purified exosome preparations. Confident that exosomes purified from the BALf of S-FLU infected mice contained viral antigens but were free of contaminating influenza virus particles, we next tested their capacity to evoke airway inflammation and drive a T cell response *in vivo*. To this end, exosomes purified from the BALf of mice infected 2 days prior with either wild-type influenza virus or S-FLU were then administered into the airways of naïve recipient animals. Consistent with our earlier findings, intranasal inoculation of S-FLU exosomes resulted in neutrophil recruitment into the airways (Figure 4C). We next tested whether exosomes generated following S-FLU infection were capable of acting as a source of antigen for the induction of an influenza virus-specific T cell response. To do this, exosomes purified from the BALf of mice infected 2 days prior with wild-type influenza virus or S-FLU were administered into the airways of naïve recipient animals and, 7 days later, the proportion influenza virus NP-specific CD8^+^ T cells were determined using an H-2D^b^-NP tetramer. Consistent with earlier experiments using wild-type influenza virus, immunization with exosomes derived from S-FLU infected mice resulted in the development of an influenza virus-specific CD8^+^ T cell response. Collectively, these results demonstrate that in the absence of any free virions, exosomes containing influenza virus antigens are capable of evoking airway inflammation and can act as a source of antigen for the development of a cellular immune response.

### Airway Exosomes Can Neutralize Influenza Virus and Block Infection of Target Cells

**Figure 5:**
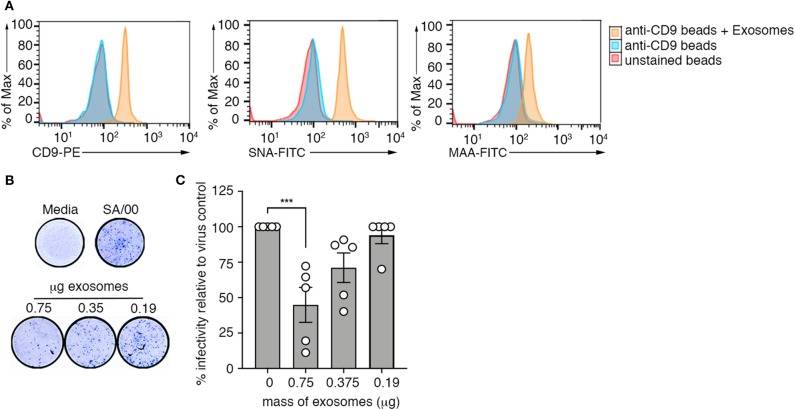
Airway exosomes can neutralize influenza virus. **(A)** Exosomes purified from the BALf of influenza infected mice were attached to magnetic beads and stained with anti-CD9-PE, MAA-FITC, and SNA-FITC and fluorescence was measured using flow cytometry. Histograms are representative of 3 independent experiments. **(B,C)** Exosomes purified from the BALf of mice that received an intranasal inoculation with an inert inflammatory stimulus (zymosan) were mixed at varying concentrations with influenza virus (SA/00) and incubated for 14 h on mouse airway epithelial cells (Let-1) under a CMC overlay before the proportion of NP^+^-foci was determined using a virospot assay. **(B)** Representative photos of virospot wells. **(C)** The percent infectivity relative to virus alone following mixing with different concentrations of purified exosomes are shown. Data pooled from 5 independent experiments (one-way ANOVA, Dunnett's multiple comparison). ***p < 0.001.

Our screen of the protein composition of exosomes released into the airways during an influenza virus infection revealed elevated expression of several mucin protein family members on airway exosomes during the acute phase of infection (Figure 1G). Previous studies have demonstrated that exosomes recovered from respiratory epithelial cell lines expressed sialylated mucins, a known receptor for influenza virus. Moreover, it was shown that these vesicles could bind and neutralize influenza virus (13). We next investigated whether exosomes generated *in vivo* also expressed sialic acids, and whether these vesicles could neutralize influenza virus infectivity. To do this, we first examined sialic acid expression on purified preparations of airway exosomes. Exosomes purified from the BALf of mice infected with influenza virus 3 days earlier were attached to anti-CD9 coated beads and then stained with phycoerythrin (PE)-tagged anti-CD9 antibody (to confirm exosome attachment), as well as fluorescein isothiocyanate (FITC)-tagged Maackia amurensis agglutinin (MAA) or Sambucus nigra agglutinin (SNA), which bind specifically to α2,3 or α2,6-linked sialic acid, respectively and examined by flow cytometry. Exosome-coated beads showed an increase in the mean fluorescence staining of anti-CD9-PE, confirming successful attachment of vesicles to the beads (Figure 5A). Moreover, both SNA and MAA bound to airway exosomes, indicating expression of α2,3 and α2,6-linked sialic acids, although the SNA signal was stronger, implying that α2,6-linked sialic acids were the more abundant linkage on airway exosomes (Figure 5A). Importantly, treatment of exosome coated beads with neuraminidase, which will act to cleave off sialic acid, reduced lectin staining which validated specificity of our lectin staining procedure (Supplementary Figure 6A).

Next, we used a microneutralisation assay to determine whether purified airway exosomes could block influenza virus infection of target cells in an *in vitro* setting. For these studies, we recovered exosomes from the BALf of mice given an inflammatory agent (zymosan) to boost exosome release into the airways. Increasing concentrations of purified exosomes incubated with a fixed concentration of influenza virus (SA/2000) were then inoculated onto Let-1 (mouse airway epithelial cell line) cell monolayers and subsequently overlaid with carboxymethcellulose to restrict viral spread. After 24 h, cell monolayers were fixed and immunostained with anti-NP antibody to detect foci of infection (Figure 5D). Figure 5E represents the percent infectivity of SA/2000 virus, calculated as the reduction in NP-foci of exosome-virus mixtures relative to the virus alone control. We observed that the number of NP-foci was significantly reduced when virus was pre-incubated with exosomes. This affect was dose dependent, as the efficiency of virus neutralization was reduced at lower concentrations of exosomes. Moreover, when we repeated the assay above, but this time treated the airway exosomes with neuraminidase to strip off surface sialic acid, virus neutralization efficiency of the exosomes was reduced, suggesting that the neutralization function of exosomes was sialic acid dependent (Supplementary Figure 6B). Collectively, these data show that sialic acid receptors are expressed on the surface of airway exosomes and that these vesicles have the ability to neutralize influenza virus, thereby preventing virus infection of target cells.

## Discussion

Here we characterized exosomes released into the airways over the course of an influenza virus infection. Our findings reveal that these vesicles dynamically change in protein composition over the course of infection, increasing expression of host proteins with known anti-influenza activity, as well as viral proteins with the potential to trigger host immune responses. This alteration in protein composition correlated with their ability to trigger pulmonary inflammation and to serve as a source of viral antigen that can be utilized for the induction of a cellular immune response. We show that α2,3 and α2,6-linked sialic acids, which represent attachment factors for influenza virus, are present on the surface of airway exosomes and that these vesicles have the ability to neutralize influenza virus infectivity. Collectively, our results highlight multiple roles for airway exosomes in antiviral defense against influenza virus.

Several recent studies have shown that cell-intrinsic antiviral pathways and interferon (IFN)-stimulated gene (ISG) signatures are upregulated in airway cells in response to influenza virus infection (19–22). As the protein composition of exosomes reflects that of the parent cell, it is not surprising that during influenza virus infection, like the airway cells, airway exosomes which originate from these cells, also adopt an antiviral protein expression profile. Here we performed a comprehensive proteomic analysis mapping the protein composition of exosomes released into the airways of mice over the course of an influenza virus infection revealing that several antiviral proteins with known anti-influenza activity, including members of the IFIT and IFITM families, were present at high levels in exosomes recovered from mice during the acute phase of infection. Others have reported similar findings *in vitro*, demonstrating that exosomes recovered from cell lines exposed to viruses or type I interferon can express an array of IFN-induced antiviral proteins (10, 23). Moreover, it was shown that antiviral protein-expressing exosomes could serve as a method for the intercellular transfer of these proteins, transmitting the antiviral effects of these proteins to neighboring cells (11, 12). Airway exosomes released during acute virus infection may amplify the interferon-induced antiviral response by serving as a method of cell-to-cell transmission of viral resistance.

Antigen presentation represents a fundamental step in the initiation of an adaptive immune response. This is the process through which antigen presenting cells, such as dendritic cells, present peptide antigens, bound to MHC class I or class II molecules, to CD8^+^ and CD4^+^ T-cells respectively, evoking their activation. Others have shown that exosomes have antigen presentation capabilities, being able to directly present antigens via classical MHC-restricted mechanisms. Exosomes secreted by murine bone marrow–derived dendritic cells carried MHC-I, MHC-II and T cell costimulatory molecules (24) and if loaded with peptide could directly activate CD8^+^ T cell responses (25). Additional studies by Thery et al. (26) show that the injection of mice with dendritic cell-derived peptide-loaded MHC-II-bearing exosomes induced the activation of antigen-specific naive CD4+ T cells *in vivo*. Moreover, exosomes released from EBV-infected B cells contained MHC II molecules that could activate specific CD4^+^ T cell clones *in vitro* (27) and exosomes released from influenza virus infected cells could serve as a vehicle for the transfer of an influenza hemagglutinin class II epitope (28). While we show here that exosomes released into the airways during the acute phase of an influenza virus infection contain viral proteins as well as MHC class I and II complexes, we find these vesicles cannot directly activate CD8^+^ or CD4^+^ T cell responses. The inability of these airway exosomes to directly prime T cell responses, despite containing viral antigen and MHC-I and MHC-II molecules, may be related to the origin of the cells in which these vesicles originate which is likely to be heterogenous. The ability to directly activate CD8^+^ and CD4^+^ T cell responses appears limited to exosomes released from professional antigen presenting cells, which in addition to higher concentrations of MHC complexes may also express other co-stimulatory molecules necessary to promote T cell activation. While airway exosomes generated during the acute phase of an influenza virus infection could not directly present viral antigen to initiate T cell activation, when cultured *in vitro* with professional antigen presenting cells, these vesicles could be utilized as a source of antigen, resulting in the activation of virus specific CD8^+^ and CD4^+^ T cells. *In vivo*, airway exosome released during an influenza virus infection are a source of viable antigen and may assist in the development of an adaptive immune response by serving as an antigen delivery system.

Exosomes released from virus-infected cells contain a variety of viral and host cellular factors that are able to modify recipient host cell responses. We demonstrated that exosomes released into the airways during the acute phase of influenza infection were able to evoke pulmonary inflammation, resulting in the generation of inflammatory cytokines and the recruitment of innate immune cells. This effect was not replicated when we immunized mice with exosomes recovered from the airways of naïve mice or animals given an inert inflammatory adjuvant, which suggests that viral components carried by exosomes were responsible for triggering the inflammatory response. Whether viral proteins or potentially viral RNA, which could also potentially be packaged within the exosome (29) was triggering pulmonary inflammation and by which signaling pathway will need to be addressed in future studies.

Here we show that exosomes released into the airways express α2,3 and α2,6-linked sialic acid, which are key receptors utilized by influenza virus to attach to target cells. We tested whether sialylated glycoproteins and glycolipids expressed on the surface of exosomes could be recognized by the influenza virus hemagglutinin (HA) glycoprotein, and in this way compete with the sialylated cell-surface receptors that are necessary for HA binding and subsequent infection of host cells. We found that airway exosomes could neutralize influenza virus infectivity. Others have shown that the intranasal delivery of sialylated nanoparticles into mice can attenuate influenza infection (30). The nanoparticles were hypothesized to act as decoy targets for influenza and thus prevent the virus from attaching to receptors on target cells; we propose that endogenous exosomes released into the airways during an influenza virus infection are likely acting via a similar mechanism. Based on these principles, the production of synthetic exosome-like nanovesicles bearing sialic acids could represent a viable therapeutic approach for the treatment of influenza virus infection. Furthermore, this approach might also be feasible for the treatment of other viruses such as coronaviruses (31) and rotaviruses (32) that also make use of sialic acid as a receptor for infection of host cells.

In summary, we demonstrate a variety of biological functions by which exosomes released into the airways during an influenza virus infection may assist in the antiviral innate immune response. Greater understanding of the antiviral properties of airway exosomes and how they modulate the course of disease may lead to the development of novel approaches that utilize exosomes, or synthetic derivatives thereof, in the treatment and management of influenza infection, which today still remains a significant threat to global health.

## Methods

### Mice and Virus Infections

C57BL/6 (CD45.2), OT-I.CD45.1, and OT-II.CD45.1 mice were bred in-house and housed in specific pathogen-free conditions in the animal facility at the Doherty Institute of Infection and Immunity, the University of Melbourne. All experiments were done in accordance with the Institutional Animal Care and Use Committee guidelines of the University of Melbourne. Mice were anesthetized with isoflurane and intranasally infected in a volume of 30 μl with 10^4^ plaque forming units (PFU) of X31-OVA_1_ (encodes the OVA_257−264_ epitope within the neuraminidase stalk) (16), 10^4^ PFU of X31-OVA_2_ (encodes the OVA_323−339_ epitope within the neuraminidase stalk) (17), or 10^6^ TCID_50_ of S-FLU (S-eGFP^*^/N1(PR8)].H1(A/PR/8/1934; pseudotyped virus based on the A/PR8/8/34 virus and modified to possess a defective HA glycoprotein, restricting it to a single round of replication (18).

### Cell Lines

Madin-Derby Canine Kidney (MDCK) cells were cultured in RPMI 1640 medium supplemented with 10% (vol/vol) heat inactivated fetal calf serum and 1% penicillin/streptomycin, 1% L-glutamine and 1% sodium pyruvate. LET1 mouse lung epithelial type I cell line were cultured in RPMI 1640 supplemented with 10% (vol/vol) heat inactivated fetal calf serum and 1% penicillin/streptomycin, 1% L-glutamine and 50 μM β-mercaptoethanol.

### Viruses

Influenza A virus X31 (H3N2) is a high yielding reassortant for the A/Aichi/2/1968 with PR8 that express the H3N2 surface glycoproteins. In addition, we used seasonal H3N2, A/South Australia/4/2000, and reverse genetics engineered X31-OVA_1_, and X31-OVA_2_. Influenza viruses were grown in 10-day embryonated chicken's eggs by standard procedures. Titres of infectious virus were determined by plaque assay on Madin-Darby canine kidney (MDCK) cells (33).

### Intranasal Delivery of Inflammatory Agents

Mice were anesthetized with isoflurane and intranasally administered in a volume of 30 μl either 200 μg of zymosan (in vivogen) or 20 μg of Poly I:C (in vivogen).

### Collection of Exosomes From Mouse Bronchial Alveolar Lavage Fluid (BALf)

Bronchoalveolar lavage was carried out by cannulating mouse tracheas with a catheter and flushing the air space with saline three times. Exosomes were purified as described in Supplementary Figure 1. Briefly, BALf was filtered through a 0.2 μm filter and in some experiments incubated with Turkey Red Blood cells (RBCs) which express saliac acids capable of binding free influenza virions in the preparation. Samples were pelleted at 800 g to remove the virus bound RBC and supernatant collected, subjected to 2 rounds of ultracentrifugation, washing the pellet in between spins with 3 ml of PBS. Exosomes were then purified by immunoabsorption using Dyanabeads FlowComp Flexi Kit following manufacturers' instructions. Briefly, FlowComp beads labeled with DSB-X Biotin conjugated α mouse-CD9 antibody were incubated overnight at 4°C with pelleted BALf after which a DynaMag-5 magnet was used to purify exosome coated beads from the supernatant. Beads were washed, and in some experiments, exosomes were then detached from the beads by the addition of a release buffer. The exosome free beads were magnetically removed and the resulting exosome containing supernatant was washed and pelleted by two rounds of ultracentrifugation. Exosomes were resuspended in PBS and the protein mass quantitated using a Standard Micro BCA Protein Assay following manufacturer's instructions. In some experiments where exosomes did not need to be released from the beads (i.e., flow cytometry analysis of exosomes), Dynabeads Biotin binder magnetic beads labeled with biotin conjugated α mouse-CD9 antibody were used in place of the FlowComp beads. In some experiments exosomes were stripped of surface sialic acids by treatment with neuraminidase from *vibrio cholerae* (100 mUnits/mL) for 30 min at 37°C. Samples were washed 3 times with PBS prior to use.

### Proteomic Analysis of Exosomes

Exosomes were purified as described above from the BALf of mice on days 0, 3, 5, 7, 10, and 20 post intranasal infection with 10^4^ PFU of x31 (H3N2). Samples were stored at −80 °C for future use. Samples were prepared for mass spectrometry analysis using the modified SP3 method as previously described (34). Briefly, exosomes were subjected to reduction/alkylation with 2 M dithiothreitol (DTT, 50 mM final concentration) for 1 h at 37°C followed by 1 M iodoacetamide (IAM, 100 mM final concentration) for 30 min in the dark at room temperature (RT). Samples were quenched with 2 M DTT (250 mM final concentration). Samples were then incubated with 18 μL of carboxylate beads (Sera-Mag Speed beads, 45152105050250, 65152105050250, GE Healthcare) and acetonitrile (ACN, 50% v/v) for 1 h. Prechilled (−20°C) acetone was added to the protein/bead mixtures at a 4 × volume (v/v) and left overnight at −20°C. Beads were washed twice with 250 μL of prechilled (−20°C) 80% acetone. Acetone was completely evaporated from the tubes using a CentriVap (Labconco) prior to addition of 20 μL of digestion buffer (10% TFE/100 mM NH_4_HCO_3_) containing Lys-C (Wako, 129-02541) and Trypsin-gold (Promega, V5280) each at a 1:50 enzyme:substrate ratio. Enzymatic digestions proceeded for 1 h at 37°C. Following the digest, samples were placed on a magnetic rack and the supernatants containing peptides were collected and an additional elution (20 μl) was performed using 2% dimethyl sulfoxide (DMSO, Sigma) prior sonication in a water bath for 1 min. The eluates were pooled together and transferred to the top of pre-equilibrated C8 StageTips (2x plugs of 3M Empore resin, #2215) for sample clean up as previously described (35). The eluates were lyophilised to dryness using a CentriVap (Labconco) prior to reconstituting in 30 μl 0.1% FA/2% ACN ready for MS analysis.

Peptides (3 μl) were separated by reverse-phase chromatography on a C18 fused silica column (I.D. 75 μm, O.D. 360 μm x 25 cm length) packed into an emitter tip (IonOpticks), using a nano-flow HPLC (M-class, Waters). The HPLC was coupled to an Impact II UHR-QqTOF mass spectrometer (Bruker) using a CaptiveSpray source and nanoBooster at 0.20 Bar using acetonitrile. Peptides were loaded directly onto the column at a constant flow rate of 400 nl/min with 0.1% formic acid in milliQ water and eluted with a 90 min linear gradient from 2 to 34% buffer B (99.9% acetonitrile and 0.1% formic acid). Mass spectra were acquired in a data-dependent manner including an automatic switch between MS and MS/MS scans using a 1.5 s duty cycle and 4 Hz MS1 spectra rate, followed by MS/MS scans at 8–20 Hz dependent on precursor intensity for the remainder of the cycle. MS spectra were acquired between a mass range of 200–2,000 m/z. Peptide fragmentation was performed using collision-induced dissociation.

Raw files consisting of high-resolution MS/MS spectra were processed with MaxQuant (version 1.5.5.1) for feature detection and protein identification using the Andromeda search engine (36). Extracted peak lists were searched against the reviewed *Mus musculus* (UniProt, July 2015) and *Influenza A* virus (strain A/X-31 H3N2) databases as well as a separate reverse decoy database to empirically assess the false discovery rate (FDR) using strict trypsin specificity, allowing up to 2 missed cleavages. The minimum required peptide length was set to 7 amino acids. In the main search, precursor mass tolerance was 0.006 Da and fragment mass tolerance was 40 ppm. The search included variable modifications of oxidation (methionine), amino-terminal acetylation, the addition of pyroglutamate (at N-termini of glutamine) and a fixed modification of carbamidomethyl (cysteine). The “match between runs” option in MaxQuant was used to transfer identifications made between runs on the basis of matching precursors with high mass accuracy (37, 38). LFQ quantification was selected, with a minimum ratio count of 2.

The time-course analysis was performed in R using the package time course (39) using the proteins' *LFQ_Intensity* values and were ranked according to the *Hotelling's T-squared statistic* resulted from the analysis. Clustering of the top 150 proteins was done in Spotfire (TIBCO) using k-means algorithm and using correlation as a distance metric (Figure 1C).

### Detection of Sialic Acid on Exosomes by Flow Cytometry

Exosomes collected from the BALf of mice were attached to magnetic beads as described above. The exosome labeled beads were stained with either Maackia amurensis agglutinin (MAA) and Sambucus nigra agglutinin (SNA) conjugated to fluorescein isothiocyanate (FITC) (EY Laboratories) diluted 1:50 in Dako Protein Block alongside α mouse CD9-PE for 15 min at room temperature. Samples were then washed 3 times in EDTA-BSS + 2% FCS and analyzed by flow cytometry.

### Electron Microscopy of Exosomes

Bead purified exosomes or as a positive control, 3 × 10^6^ PFU X31 (H3N2) allantoic fluid were fixed in 2% paraformaldehyde overnight at 4°C. Carbon-coated copper grids were treated with 1% Alcian blue for 5 min and then washed in dH_2_O. Fixed exosomes and virus preparations were placed onto grids and allowed to adhere for 1 min before excess liquid was removed by blotting. Grids were transferred to a droplet of 0.05M glycine for 10 min then blocked in PBS + 0.1% bovine serum albumin (BSA) for 30 min. Grids were stained with primary rat α mouse CD9 antibody overnight at 4°C, washed in PBS + 0.1% BSA and then stained for 1 h at room temperature with secondary α Rat IgG-gold antibody diluted 1:100 in PBS + 0.1% BSA. Grids were then washed five times in PBS with 0.1% BSA for 5 min. Grids were negatively stained with ammonium molybdate diluted 1.5% (vol/vol) in water for 30 s and viewed using a Tecnai Spirit electron microscope at 120 kV.

### Adoptive Transfer and T Cell Isolation

Naïve OT-I or OT-II T cells were purified from the lymph nodes and spleen of OT-I or OT-II TCR transgenic mice, as previously described (40). Briefly, cells were purified after a negative depletion step using antibodies against CD11b (M1/70), F4/80, Ter-119, Gr-1 (RB6), MHC class II (M5/114), and either CD4 (GK 1.5) for OT-I enrichment or CD8 (YTS 169.4) for the OT-II purification followed by incubation with anti-rat IgG-coupled magnetic beads (Dynal Biotech) following the manufacturer's protocols. T cell preparations were 90–95% pure as determined by flow cytometry. 2 × 10^6^ purified CD8 OT-I.CD45.1 or CD4 OT-II.CD45.1 T cells were labeled with 5 μM CFSE (Sigma-Aldrich) prior to intravenous injection into mice.

### *In vivo* Exosome Intranasal Administration

Purified exosomes (0.75 μg) recovered from the BALf of donor mice were administered intranasally to anesthetized recipient mice in a volume of 30 μl. In some experiments, exosomes were labeled with PKH26 lipophilic membrane dye prior to intranasal delivery following manufactures' instructions.

### Flow Cytometry

Single cell suspensions were prepared from mediastinal lymph node by mechanical disruption. Mice were perfused prior to the harvest of the lung tissue which was enzymatically digested for 1 h at 37°C in 3 mL of collagenase type 3 (3 mg/mL in RPMI 1640 supplemented with 2% FCS). Cells were stained for 25 min on ice with the appropriate mixture of monoclonal antibodies and washed with PBS with 1% BSA. The conjugated monoclonal antibodies were obtained from BD Pharmingen or Biolegend include: anti-CD8 (53-67), anti-CD4 (GK1.5), anti-Va2(B20.1), anti-MHC-II (M5/114), anti-CD11c (N418), anti-CD45.1 (A20), anti-Ly6g (1A8), anti-CD11b (M1/70). H2-Db-NP_366_, tetramer was made in-house.

### Antigen Presentation Assay

Total DCs were isolated from spleen tissue that was enzymatically digested with collagenase III and DNAse as previously described (41). In brief, splenic cells were digested with Dnase I (Boehringer-Manheim, Mannhein, Germany) and collagenase III (Worthington Biochemicals, Freehold, NJ) and enriched for light-density cells by centrifugation in 1.077 g/cm^3^ Nycodenz (Nycomed Pharma, Oslo, Norway). Cells were isolated after a depletion step using antibodies against CD3 (KT3-1.1), Thy-1 (T24/31.7), Ter 119, Ly6G (RB68C5), and CD45R (RA36B2), followed by incubation with anti-rat IgG-coupled magnetic beads (Dynal, Oslo, Norway) following the manufacturer's protocol. Spleen DC were further purified by sorting CD11c+MHC-II+ F4/80- populations on an Aria III.

Purified exosomes (0.75 μg/well) were added to 96-well plates with CFSE-labeled OT-I or OT-II cells (5 × 10^4^) with or without 10^4^ spleen DCs and were cultured at 37°C in RPMI 1640 medium supplemented with 10% FCS, 50 μM 2-ME, 2 mM L-glutamine, 100 U/ml penicillin, 100 μg/ml streptomycin, and 0.5 mM CpG. T cell proliferation, as measured as a loss of CFSE, was analyzed by flow cytometry after 60–65 h of culture.

### *In vitro* Assessment of Viral Infection

MDCK cells (4 x 10^4^ cells/well) in a 96 well flat-bottom plate were cultured in serum free media for 1 h at 37°C with either crude BALf, purfied exosome samples, or influenza virus at specific multiplicity of infection (moi). Cells were washed to remove free virus and cultured at 37°C in RPMI with 10% FCS for 12–24 h. Cells were fixed in BD Cytofix/Cytoperm and stained for intracellular influenza virus nuclear protein (α NP-FITC, abcam). The proportion of cells staining positive for α NP-FITC was then measured by flow cytometry.

### Assessment of Cytokine Production in BALf

Bronchoalveolar lavage was carried out by cannulating mouse tracheas with a catheter and flushing the air space with saline three times. The levels of IFN-γ, TNF-α, IL-6, IL-10, IL-12p70, and MCP-1 in the BALf was determined using a BD Cytometric Bead Array Mouse Inflammation Kit (BD Biosciences, San Diego, CA, USA) according to the manufacturer's instructions. Samples were then analyzed on a Becton Dickinson FACSCanto flow cytometer (Franklin Lakes, NJ, USA) and data analyzed using FlowJo software (Tree Star, Inc., Ashland, OR, USA). In some experiments a cytokine/chemokine concentrations in bronchial alveolar lavage fluid (BALf) was measured using a LEGENDplex mouse anti-virus response standard panel (Biolegend) following manufacturer's instructions.

### Virospot Microneutralisation Assay

Varying concentrations of purified exosomes were mixed with a fixed concentration (50–200 virospots) of A/South Australia/4/2000 Influenza A virus and incubated at 37°C for 1 h. The virus-exosome mixture, or as a control, virus alone were overlaid onto a monolayer of Let-1 cultured at 4 × 10^4^ cells/ well in a 96 well flat-bottom plate. After 1 h a carboxymethcellulose (CMC) overlay, generated by mixing 2XDMEM 1:1 with 6.4% (w/v) CMC diluted in distilled water was added to restrict viral spread. Plates were incubated overnight at 37°C. The overlay was then removed, the cells were fixed in 80% (v/v) of acetone at 4°C, blocked in 5% skim milk powder diluted in 0.05% PBS tween prior to being stained with mouse anti-influenza A virus nuclearprotein (NP, CSL Pty Ltd.) followed by secondary anti-mouse-horse radish peroxidase. TrueBlue Peroxidase substrate was added, and the wells imaged using a CTL ImmunoSpot analyser.

### RT-PCR for Influenza vRNA

The levels of viral RNA (vRNA) or influenza A virus matrix (M) gene were determined by qRT-PCR. Briefly, vRNA was extracted from purified exosome preparations using the QIAamp viral RNA minikit (Qiagen) according to the manufacturer's instructions. To determine the copy numbers of the IAV M segment, 4 μL of vRNA were amplified using specific primers and probe in combination with the SensiFAST™ Probe Lo-ROX One-Step Kit (Bioline, Australia). The qRT-PCR reaction was carried out using the Applied Biosystems® QuantStudio™ 7 Flex Real-Time PCR System. Specific primer sequences are available upon request. vRNA copy numbers were calculated by generating a standard curve using serial dilutions of plasmid containing DNA for the IAV Matrix gene from A/California/7/2009.

## Data Availability Statement

The mass spectrometry proteomics data have been deposited to the ProteomeXchange Consortium via the PRIDE (42) partner repository with the dataset identifier PXD018389.

## Ethics Statement

The animal study was reviewed and approved by The Animal Ethics Committee at the University of Melbourne.

## Author Contributions

LW, JB, and PR designed the project. Experiments and data analysis was performed by LW, JB, GI, LD, FV-L, MZ, and VB-W. LW and PR contributed to writing the manuscript and editing of the manuscript.

## Conflict of Interest

The authors declare that the research was conducted in the absence of any commercial or financial relationships that could be construed as a potential conflict of interest.
