# Supplementary material for: Airway Exosomes Released During Influenza Virus Infection Serve as a Key Component of the Antiviral Innate Immune Response
Source: Front Immunol. 2020 May 12;11:887. doi: 10.3389/fimmu.2020.00887 (PMC7236881; doi:10.3389/fimmu.2020.00887)
Supplement: Supplementary file 1 [file Data_Sheet_1.DOCX]

Supplementary Material

Supplementary Table 1: Proteins detected in airway exosomes over the course of an influenza virus infection

Supplementary Table 2: Proteins elevated in airway exosomes days 3-5 post infection. Related to Fig 1d

Supplementary Table 3: Proteins elevated in airway exosomes days 3-7 post infection. Related to Fig 1e


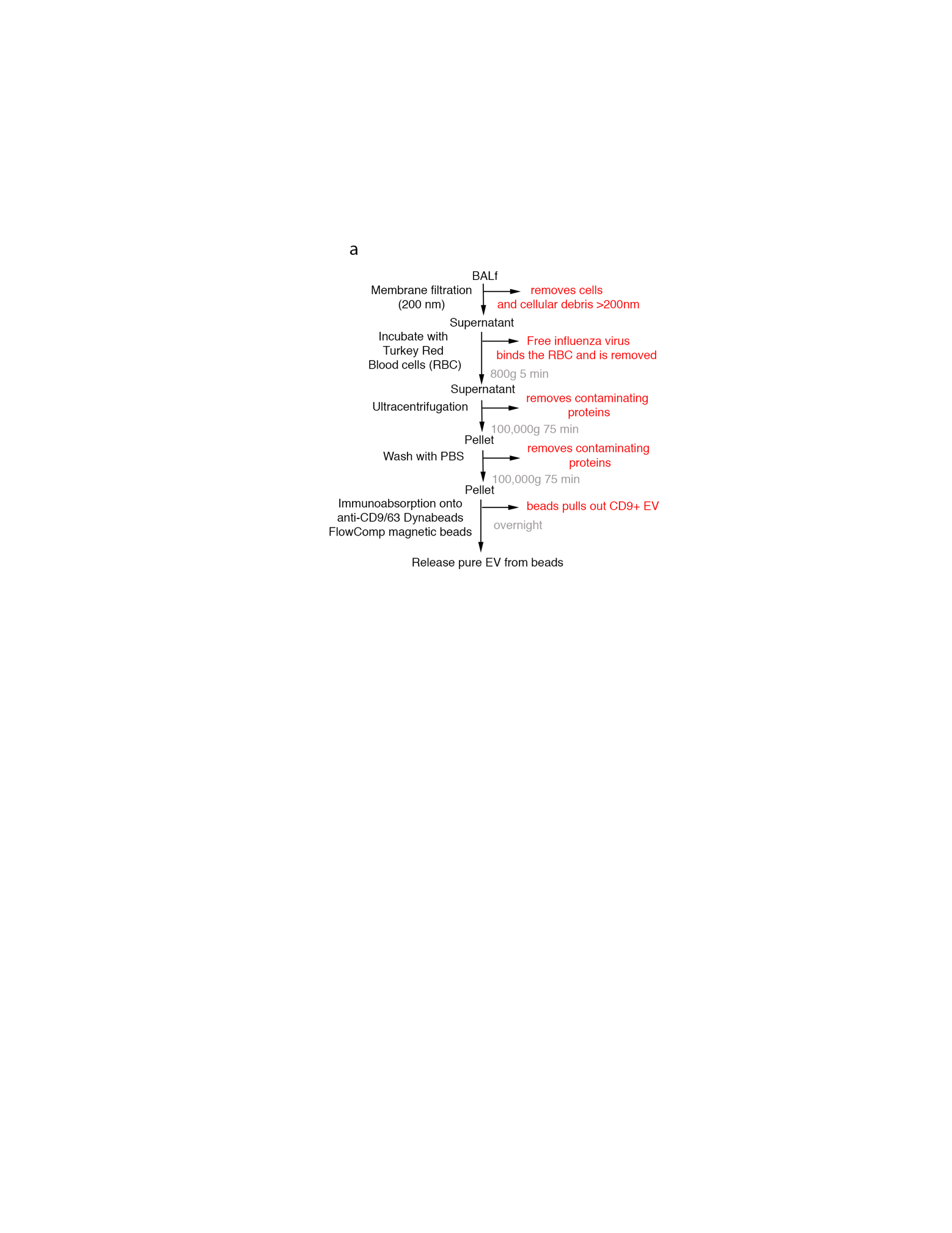


**Supplementary Figure 1**: Schematic diagram of the methodology used to purify exosomes from the BALf of mice


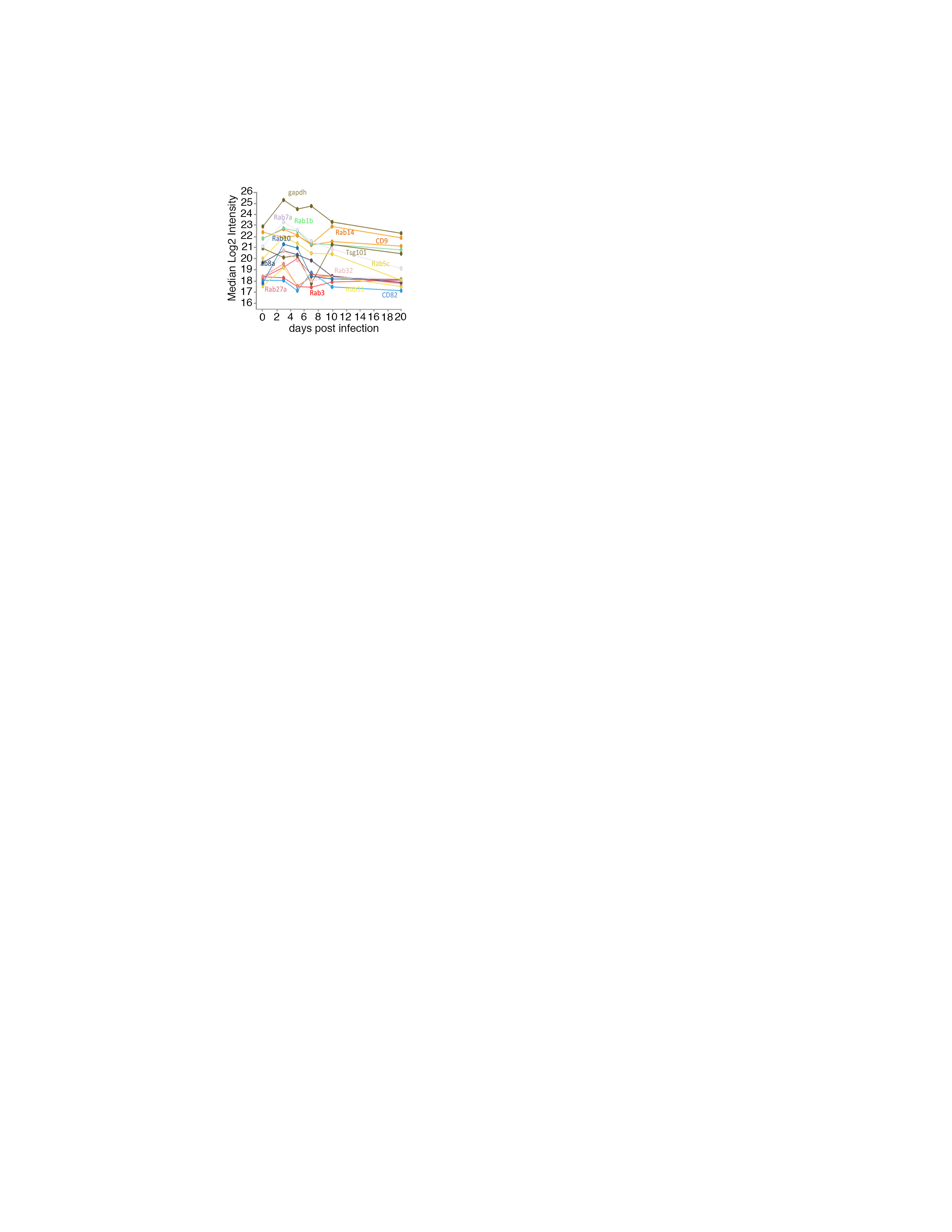


**Supplementary Figure 2:** Median Log2 Intensity of proteins known to be highly enriched in exosomes.

**Supplementary Figure 3: Airway exosomes from influenza virus-infected mice evoke airway inflammation**

Levels of inflammatory cytokines in mouse BALf. Mice that received 0.75 μg of airway exosomes recovered from mice 2 days after intranasal delivery of influenza virus were killed 4 days later for analysis of inflammatory cytokines in BALf. In these studies, BALf of naïve mice and from mice infected 4 days earlier with influenza virus were included as controls. Data pooled from 2 experiments. Symbols represent individual mice (n=4-7 mice per group, one-way ANOVA Dunnett’s multiple comparison).

**Supplementary Figure 4: Airway exosomes contain influenza viral RNA**

The amount of influenza viral RNA present in either of purified exosomes or matched amounts of crude BALf recovered from mice 2 days post intranasal influenza virus infection. The levels of influenza vRNA for the influenza A Matrix (M) gene segment was determined via qRT-PCR. Data are expressed as the mean copy number/ml + sem. Data pooled from 3 independent experiments.


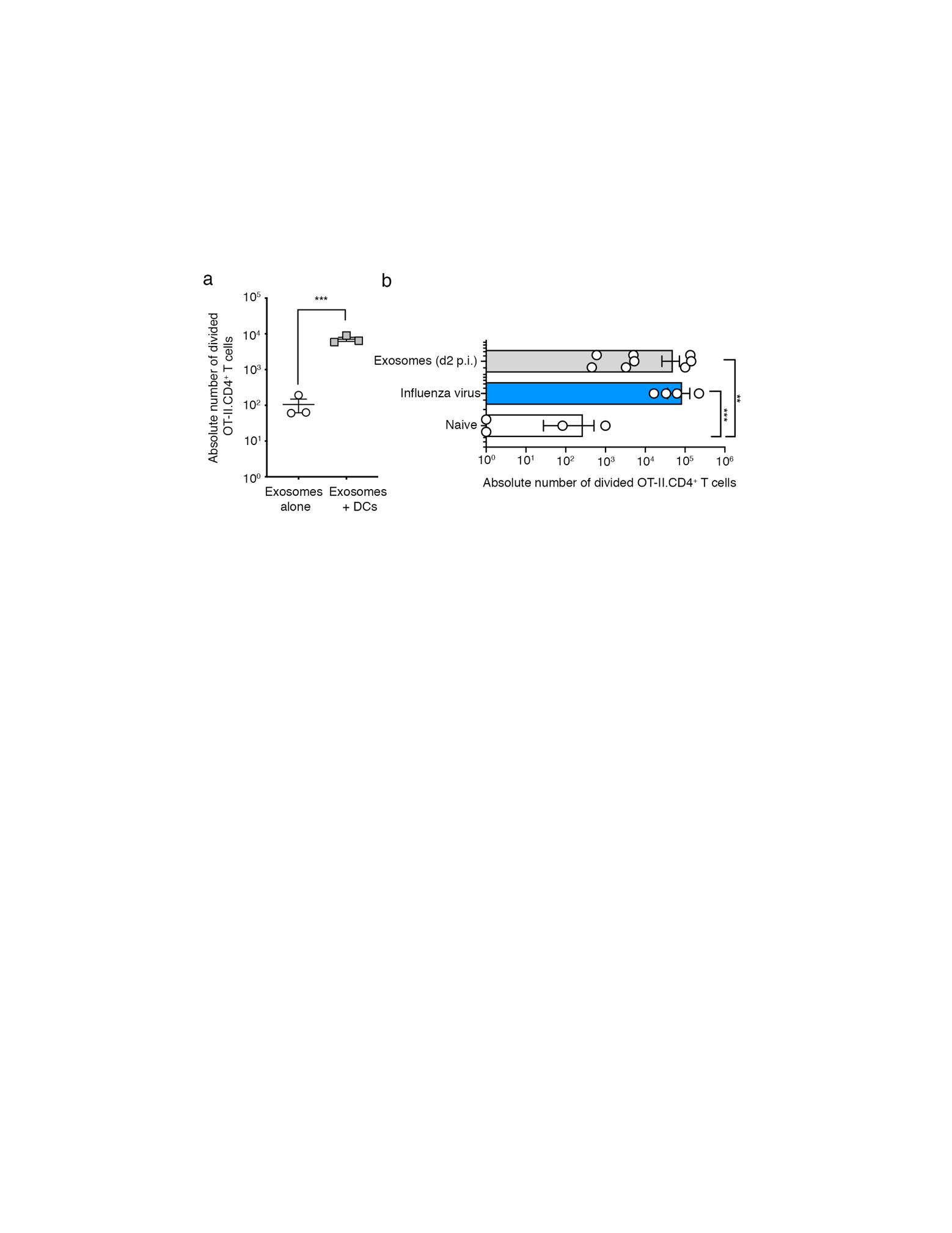


**Supplementary Figure 5: Exosomes from the BALf of influenza virus infected mice can serve as a source of MHC-II antigen.**

(a) Exosomes purified from the BALf of mice infected 2 days prior with 10^4^ PFU of x31-OVA_2_ were cultured with 5 x10^4^ CFSE-labelled OT-II CD4^+^ T cells with or without 1 x10^4^ DCs. T cell proliferation was measured 60 hrs later. The absolute number of divided OT-II.CD4^+^ T cells is shown. Data pooled from 3 independent experiments. Symbols represent the mean+sem (t-test)

(b) Mice injected with 2 x10^6^ CFSE labelled OT-II.CD4^+^ T cells were intranasally administered either saline (naïve) or x31-OVA_2_ or 0.75 μg of exosomes recovered from the BALf of mice infected with x31-OVA_2_ two days earlier and the absolute number of divided OT-II cells in the mLN was measured 4 days later. Data pooled from 4 independent experiments (n=4-8 mice per group, one way ANOVA, Dunnett’s multiple comparison).


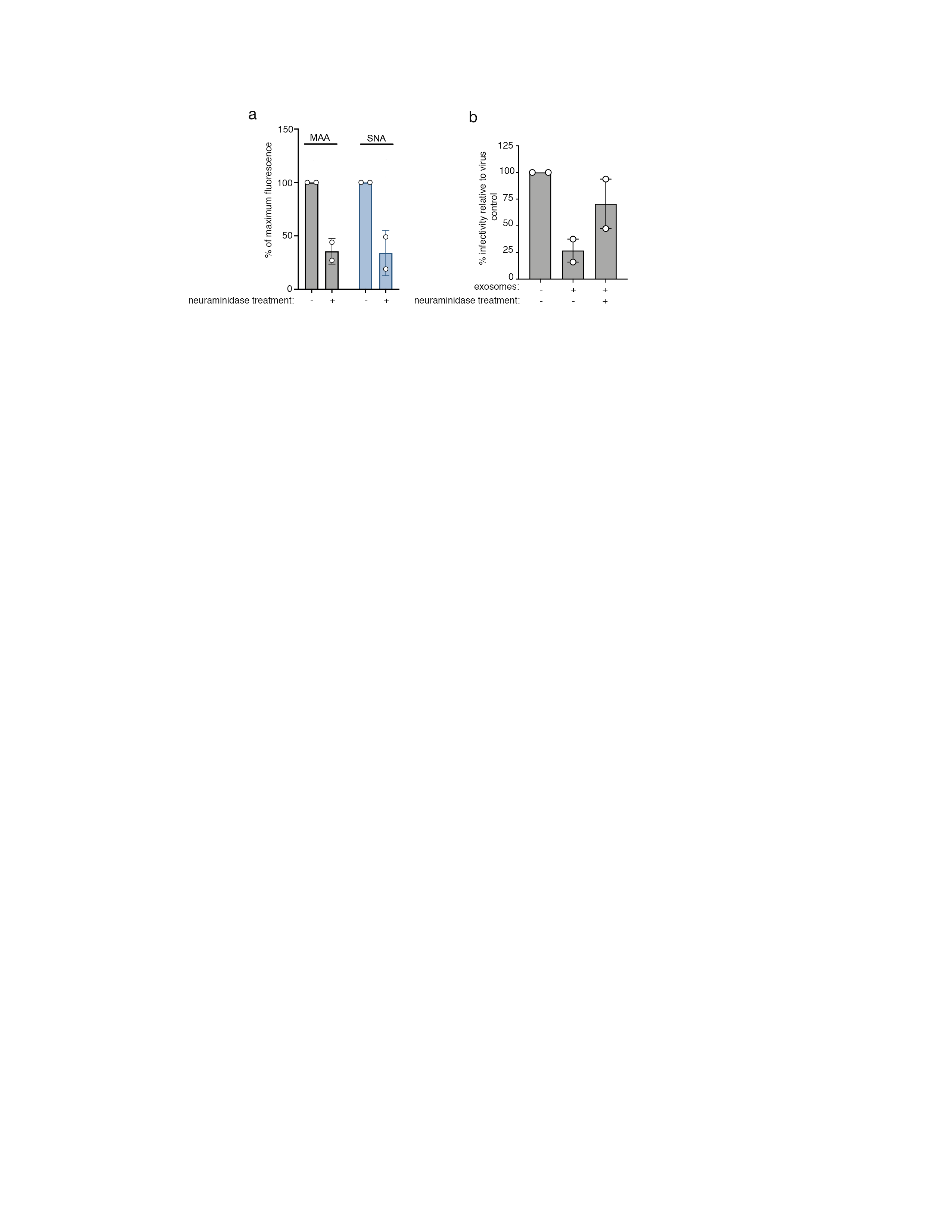


**Supplementary Figure 6: Neuraminidase treatment of exosomes reduces lectin staining fluorescence and neutralisation ability**

(a) Exosomes purified from the BALf of influenza infected mice were attached to magnetic beads and treated with neuraminidase (100 mUnits/mL for 30 minutes at 37^o^ C) or mock treated. Beads were washed and stained with MAA-FTIC and SNA-FTC and fluorescence was measured using flow cytometry. Graph represents the percentage of maximum fluorescence relative to mock treated exosomes. Data pooled from 2 independent experiments.

(b) 0.75 μg of exosomes purified from the BALf of mice that received an intranasal inoculation with an inert inflammatory stimulus (zymosan) were treated with neuraminidase (100 mUnits/mL for 30 minutes at 37^o^ C) or mock treated prior to mixing with influenza virus (SA/00). Preparations were incubated for 14 h on mouse airway epithelial cells (Let-1) under a CMC overlay before the proportion of NP^+^-foci was determined using a virospot assay. The percent infectivity relative to virus alone are shown. Data pooled from 2 independent experiments.
